# Editorial: Applications of artificial intelligence, machine learning, and deep learning in plant breeding

**DOI:** 10.3389/fpls.2024.1420938

**Published:** 2024-05-22

**Authors:** Maliheh Eftekhari, Chuang Ma, Yuriy L. Orlov

**Affiliations:** ^1^ Department of Horticultural Sciences, Faculty of Agriculture, Tarbiat Modares University, Tehran, Iran; ^2^ State Key Laboratory of Crop Stress Resistance and High-Efficiency Production, Center of Bioinformatics, College of Life Sciences, Northwest A&F University, Xianyang, Shaanxi, China; ^3^ Key Laboratory of Biology and Genetics Improvement of Maize in Arid Area of Northwest Region, Ministry of Agriculture, Northwest A&F University, Xianyang, Shaanxi, China; ^4^ Systems Biology Department, Institute of Cytology and Genetics Siberian Branch of the Russian Academy of Sciences (SB RAS), Novosibirsk, Russia; ^5^ Agrarian and Technological Institute, Patrice Lumumba Peoples’ Friendship University of Russia, Moscow, Russia; ^6^ Chair of Information and Internet Technologies, Institute of Biodesign and Complex System Modelling, Sechenov First Moscow State Medical University of the Ministry of Health of the Russian Federation (Sechenov University), Moscow, Russia

**Keywords:** AI-driven plant breeding, omics data analysis, crop resilience, disease detection, global food security

In recent years, the field of plant breeding has witnessed a paradigm shift driven by advancements in artificial intelligence (AI) technologies, including machine learning (ML) and deep learning (DL) technologies. These cutting-edge techniques have transformed our understanding of plant biology. From decoding the intricate molecular mechanisms of plant defense to automating disease detection and optimizing nutrient levels, AI is reshaping the landscape of plant breeding (Hamazaki and Iwata, 2024). AI-assisted omics techniques offer insights into plant-pathogen interactions and facilitate the identification of stress-responsive genes (Mahmood et al., 2022; Chao et al., 2023).

This Research Topic presents 16 papers on the application of computer techniques in plant science. Murmu et al. highlighted the potential of AI algorithms, particularly ML and DL, in decoding complex omics data to elucidate the molecular foundations of plant defense. In their review article, they explored AI-assisted omics techniques’ applications, challenges, and prospects in enhancing crop protection strategies and ensuring global food security amidst environmental challenges. By integrating AI with omics technologies, researchers can unravel intricate gene regulatory networks and develop targeted interventions for enhancing crop resilience.

As we confront the challenges of climate change and emerging diseases, AI-driven approaches offer a robust toolkit for ensuring global food security and sustainability in agriculture. Climate change poses significant threats to agricultural systems, emphasizing the importance of elucidating cold defense mechanisms in crops. Konecny et al. introduced the Self Organizing Maps (SOM)-based ML method to decipher gene expression patterns in response to different temperature regimes. Their study accentuated the value of SOM as a promising tool for unraveling complex transcriptomic data and provided insights into the molecular basis of cold defense mechanisms in grapevines.

Genomic selection has revolutionized plant breeding by enabling the prediction of breeding values based on genomic information. Despite significant advancements, accurately measuring the long-term genetic value remains challenging. The concept of oracle selection offers a unique perspective on the challenges and opportunities in plant breeding. Vanavermaete et al. discussed the implications of oracle selection for breeding program optimization and highlighted its potential to drive innovation in genomic selection methodologies. While not directly applicable in practical scenarios, oracle selection stimulates critical thinking and fosters innovation in breeding program design.

Phenomics has emerged as a vital tool for bridging the genotype-phenotype gap in plant breeding. Singh et al. addressed the challenge of predicting biomass accurately using models developed from RGB images, emphasizing the need for stable performance across experiments. The study’s findings simplify uncovering novel genes related to biomass production and breaking the yield plateau through non-invasive, high-throughput phenotyping techniques.

ML algorithms, such as random forests (RFs) and XGBoost, have demonstrated exceptional predictive accuracy in estimating crop yield and identifying desirable genotypes. Pugh et al. showed the efficacy of ML models in predicting peanut yield and enhancing breeding efficiency using high-throughput phenotyping data obtained from unmanned aerial vehicles. By applying above-ground traits to estimate underground yield, their approach circumvents traditional limitations in phenotyping.

In this regard, Li et al. highlighted the advancements in unmanned aerial remote sensing and vegetation indices for winter wheat yield prediction but emphasized the need for effective feature selection to enhance model performance. Their findings prove the superiority of the Cubist model, showing the efficacy of the PCRF-RFE method and providing valuable insights for future research in yield prediction and feature selection. Mousavi et al. addressed the yield prediction challenge by integrating soil and environmental factors with ML to predict wheat yield. By integrating soil properties, topographic attributes, and vegetation indices, their study applied RF and artificial neural networks (ANNs) to map actual wheat yield, highlighting the potential of ML in optimizing agricultural production.

DL methods, particularly convolutional neural networks (CNNs), have revolutionized image-based analysis in plant breeding. Davidson et al. present a groundbreaking study that explores the untapped potential of CNNs in automating the analysis of mature somatic embryos, a critical process in plant propagation. By employing CNNs for semantic and instance segmentation of conifer embryos, their study confirmed the precision and efficiency of neural network-based methods in delineating morphological regions and counting cotyledons, providing unprecedented accuracy and efficiency compared to previous techniques. By enabling precise segmentation and counting of morphological features, AI-assisted approaches open avenues for further analysis of somatic embryos and enhancing crop productivity and sustainability in agriculture and forestry.

Pubescence, a key phenotypic trait in plants, correlates with stress resistance, particularly in wheat. Visual determination of glume pubescence aids in cultivar selection but is subjective and laborious. Artemenko et al. proposed an AI-driven approach using CNNs to automate glume pubescence detection, addressing the limitations of traditional methods and enhancing breeding efficiency. They employed image segmentation to extract spike contours, followed by cropping for uniformity. Investigation into image scale and distortions revealed optimal conditions for accurate pubescence prediction. Their method offers a reliable and efficient solution for phenotype analysis, empowering breeders with advanced tools for cultivar selection and stress resilience enhancement.

Deep learning models have the potential to optimize agricultural practices and enhance crop management strategies. Wheat cultivation faces the recurring issue of seedling shortages and damage in agricultural fields, leading to reduced grain yields and economic losses. Feng et al. offered valuable insights into the application of DL for wheat seedling variety recognition, introducing the MssiapNet model as a promising solution for addressing challenges in agricultural productivity. Their findings highlighted the importance of using advanced technologies to streamline variety identification processes and improve crop management practices in wheat cultivation. SCGNet, another novel DL model, introduced by Sun et al. incorporates several modules designed to enhance information exchange and feature multiplexing. It is tailored for rapid and efficient varietal classification of wheat grains. By employing DL techniques, they demonstrated the feasibility of efficient and accurate varietal identification in plant breeding.

A combination of DL models i.e., one-dimensional CNN (1D-CNN) model with a hyperspectral imaging system was employed by Li et al. for predicting cottonseed vitality. They extracted relevant information for cottonseed vitality prediction through preprocessing techniques and feature extraction algorithms. By indicating the efficacy of the 1D-CNN model in predicting cottonseed vitality, their study facilitated the development of automated detection devices, revolutionizing cottonseed quality assessment practices. Moreover, the fusion of spectral and image features enhanced prediction accuracy, offering a comprehensive approach to cottonseed vitality evaluation.

The integration of near-infrared hyperspectral imaging and transfer learning holds great promise for advancing seed vigor detection and enhancing agricultural productivity. Qi et al. offered a pioneering methodology for detecting rice seed vigor using near-infrared hyperspectral imaging and transfer learning techniques. Their findings provide valuable insights into optimizing crop seed quality assessment processes, thereby improving rice yield and quality. Their findings showed the efficacy of the MixStyle transfer strategy in improving the generalization ability of CNN models across different rice varieties, leading to the rapid and accurate assessment of seed vigor. This approach has profound implications for enhancing rice production efficiency.

Identifying pests and diseases affecting plant crops is a laborious and error-prone task, often leading to suboptimal control measures and decreased yields. By accurately identifying and categorizing plant diseases, AI technology enables breeders to select and develop disease-resistant plant varieties more efficiently. Disease-resistant crops are essential for sustainable agriculture, as they reduce the reliance on chemical pesticides and contribute to higher yields and food security. Therefore, advancements in plant disease detection through AI-driven methods directly support plant breeding initiatives aimed at developing resilient and high-yielding crop varieties.

The YOLO (You Only Look Once) architecture, known for its real-time object detection capabilities, is employed for object detection in plant image analysis (Liu et al., 2024). Li et al. showed the efficacy of the CFNet-VoVGCSP-LSKNet-YOLOv8s model in accurately identifying cotton pests and diseases amidst challenging environmental conditions. The model’s superior performance offers a promising solution for real-time monitoring and early intervention in pest and disease outbreaks, thereby mitigating yield losses and reducing reliance on chemical interventions. This article heralds a new era in cotton plant breeding, wherein cutting-edge AI, ML, and DL techniques converge to address age-old challenges with remarkable precision and efficiency. By providing a robust technical foundation for pest and disease identification and control, the CFNet-VoV-GCSP-LSKNet-YOLOv8s model emerges as a game-changer in the quest for agricultural sustainability. On the other hand, Ullah et al. introduced a novel DL-based architecture, DeepPlantNet, for efficient and accurate prediction and categorization of plant leaf diseases. With 28 learned layers including convolutional and fully connected layers, DeepPlantNet achieved high accuracy in classifying various plant diseases into multiple categories. Their results indicated AI potential to significantly reduce agricultural losses by aiding in timely disease identification, demonstrating superiority over existing methods.

Efficient optimization of nutrient levels enhances crop quality and resilience, attributes vital for successful plant breeding programs aiming to develop high-yielding and resilient crop varieties. Cho et al. introduced a decision-tree-based dosing algorithm for managing ion-specific nutrient solutions in closed hydroponic systems, crucial for crop quality and nutrient recycling efficiency. Evaluating its performance, the algorithm demonstrated feasible accuracies and significantly reduced fertilizer injections and carbon emissions, promising more sustainable agricultural practices that align with the broader goals of environmentally conscious plant breeding initiatives.

Overall, AI, ML, and DL techniques offer unique opportunities from deciphering complex omics data to automating phenotypic trait analysis and disease detection to revolutionize breeding practices, develop stress-tolerant and high-yielding crop varieties, and contribute to global food security in the face of escalating environmental challenges (Orlov and Chen, 2023; Ngugi et al., 2024). Continued investment in AI applications in plant breeding holds the key to unlocking the full potential of agriculture and ensuring a prosperous and sustainable future for generations to come.

We aim to continue a series of Research Topics (special journal issues) on computational plant biology and bioinformatics application (Orlov and Baranova, 2020; Anashkina et al., 2023) in *Frontiers*.

## Author contributions

ME: Conceptualization, Writing – original draft, Writing – review & editing. CM: Writing – original draft, Writing – review & editing. YO: Writing – original draft, Writing – review & editing.
